# LncRNA MALAT1 Modulates TGF-β1-Induced EMT in Keratinocyte

**DOI:** 10.3390/ijms222111816

**Published:** 2021-10-30

**Authors:** Liping Zhang, Junyi Hu, Bahar I. Meshkat, Kenneth W. Liechty, Junwang Xu

**Affiliations:** 1 Department of Physiology, College of Medicine, University of Tennessee Health Science Center, Memphis, TN 38163, USA; lzhan112@uthsc.edu (L.Z.); bmeshkat@uthsc.edu (B.I.M.); 2 Laboratory for Fetal and Regenerative Biology, Department of Surgery, University of Colorado Denver—Anschutz Medical Campus and Children’s Hospital Colorado, Aurora, CO 80045, USA; JUNYI.HU@CUANSCHUTZ.EDU (J.H.); Kenneth.Liechty@childrenscolorado.org (K.W.L.)

**Keywords:** diabetic wounds, long non-coding RNA, MALAT1, epithelial mesenchymal transition

## Abstract

One of the major complications in diabetes is impaired wound healing. Unfortunately, effective therapies are currently lacking. Epithelial to mesenchymal transition (EMT) is a critical process involved in cutaneous wound healing. In response to injury, EMT is required to activate and mobilize stationary keratinocytes in the skin toward the wound bed, which allows for re-epithelialization. This process is stalled in diabetic wounds. In this study, we investigate the role of long non-coding RNA (lncRNA), MALAT1, in transforming growth factor beta 1(TGF-β1)-induced EMT of human keratinocyte (HaCaT) cells. Initially, we detected MALAT1 and TGF-β1 expression in non-diabetic and diabetic wounds and found that these expression are significantly up-regulated in diabetic wounds. Then, HaCaT cells were cultured and exposed to TGF-β1. The EMT of HaCaT cells were confirmed by the increased expression of CDH2, KRT10, and ACTA2, in addition to the down-regulation of CDH1. Knockdown of MALAT1 was achieved by transfecting a small interfering RNA (SiRNA). MALAT1 silencing attenuates TGFβ1-induced EMT. Mechanistically, MALAT1 is involved in TGF-β1 mediated EMT through significantly induced ZEB1 expression, a critical transcription factor for EMT. In summary, lncRNA MALAT1 is involved in TGFβ1-induced EMT of human HaCaT cells and provides new understanding for the pathogenesis of diabetic wounds.

## 1. Introduction

Diabetes has reached pandemic proportions worldwide. It is currently estimated that the number of diabetic patients will reach approximately 700 million by the year 2045 [1]. Diabetic complications, including that of impaired wound healing, represent a significant medical problem in society today. In fact, the annual cost of diabetic lower extremity ulcers alone exceeds 1.5 billion dollars annually [2]. More than one-third of diabetic patients suffer from diabetic foot ulcers(DFU) [1], which are known to be the number one cause of non-traumatic lower extremity amputation. Despite the enormous impact of these chronic wounds on both individuals and society, effective therapies are unfortunately lacking at the current time.

Cutaneous wound healing is a complex, multi-step process involving overlapping stages of hemostasis, inflammation, re-epithelialization, granulation tissue formation, neovascularization, and remodeling. The re-epithelialization is meant to restore the epidermal barrier. During this process, the morphology of keratinocytes temporarily changes to a spindle morphology at the wound edge, which is thought to occur due to an epithelial–mesenchymal transition (EMT). A defining characteristic of EMT is the loss of epithelial phenotypes and cell polarity as well as the acquisition of a mesenchymal phenotype, including attenuation of E-cadherin (E-cad), and acquisition of N-cadherin (N-cad), α-SMA and cytokeratin10 [3]. TGF-β1 has multiple implications in wound healing and is a major cytokine-inducing EMT [4].

Long non-coding RNAs (lncRNAs) are non-protein coding RNAs with a length of more than 200 nucleotides. LncRNAs are able to regulate gene expression through epigenetic, transcriptional, post-transcriptional, translational and post-translational level by participating in physiological and pathological processes in human diseases [5]. Recent studies indicate that lncRNAs play a role in diabetic wound healing [6,7,8,9]. The metastasis-associated lung adenocarcinoma transcript 1 (MALAT1), also known as nuclear-enriched abundant transcript 2 (NEAT2), is a large, infrequently spliced non-coding RNA. Recent studies have suggested that the lncRNA MALAT1 is involved in the process of EMT in cancer cells through a variety of mechanisms [10,11,12,13,14,15,16,17,18,19,20,21]. Moreover, emerging evidence has revealed a potential contribution of MALAT1 to the development of diabetic complications [22,23,24,25]. However, to the best of our knowledge, whether MALAT1 can regulate EMT of skin keratinocyte and contribute to the pathogenesis of diabetic wounds has not been fully elucidated. Thus, we asked whether MALAT1 contributes to EMT in diabetic wounds. In this study, we aimed at exploring the role of MALAT1 in TGF-β1 mediated EMT of keratinocyte.

## 2. Materials and Methods

### 2.1. Animal Studies

All experimental protocols were approved by the Institutional Animal Care and Use Committee at University of Tennessee Health Science Center (Memphis, TN, USA), following the guidelines described in the NIH Guide for the Care and Use of Laboratory Animals. Ten-week-old age-matched, female, genetically diabetic C57BKS.Cg-m/Leprdb/J (Db) mice and heterozygous, non-diabetic (non-Db), female controls were obtained from Jackson Laboratory (Bar Harbor, ME, USA) and used in the following experiments. Prior to wounding, the posterior neck and back were shaved and depilated. The area was cleared with an alcohol swab and a single dorsal full-thickness wound was made with an 8 mm punch biopsy (Miltex, Inc. Davies Drive, York, PA, USA). All wounds were performed under inhaled anesthesia with Isofluorane and then dressed with a Tegaderm (3M), which was subsequently removed on post-operative day 2. A full-thickness skin sample, centered on the wound, was harvested 3 and 7 days after surgery (n = 5 per timepoint).

### 2.2. Cells Culture Conditions and Treatments

HaCaT cells, obtained from ATCC, were cultured in full medium comprising Dulbecco’s modified eagle high-glucose (DMEM, Sigma-Aldrich, St. Louis, MO, USA) supplemented with 10% fetal bovine serum (FBS; Gibco, Waltham, MA, USA) and maintained at 37 °C in a humidified atmosphere containing 5% CO_2_. For further experiments, cells were seeded in 6-well plates and cultured for 12 h. Thereafter, cells were starved for 16 h and were stimulated with 20 ng/mL TGFβ1 (PeproTech, Rocky Hill, NJ, USA). SiRNA transfection of HaCaT cells was performed using the Lipofectamine 2000 reagent (Invitrogen, Life Technologies, Carlsbad, CA, USA) according to the manufacturer’s instructions. For overexpression, HaCaT cells were transfected with pMALAT1 or pcDNA3.1 control. Transfection reagents mimic antagomirs and control miRNAs were purchased from Invitrogen. 24 h following transfection, the cells were processed for gene expression analysis.

### 2.3. Immunocytochemistry

HaCaT cells were cultivated in the presence of a high concentration of glucose, with or without TGF-β1, on a 24-well plate cover glasses. Twenty-four hours later, cells were fixed with 4% paraformaldehyde for 15 min, then permeabilized with 0.1% Triton X-100 in PBS. After washing with PBS, cells were preincubated in blocking solution (1% bovine serum albumin, BSA) in PBST for 1 h at room temperature and incubated with primary mouse antibody against Keratin 10(KRT10) (ab76318, Abcam, Waltham, MA, USA) overnight. After rinsing with PBS, the cells were incubated with a second anti-mouse antibody labeled with Alexa 488 (Abcam, Waltham, MA, USA) for 1 h. Nuclei were stained with 40,6-diamidino-2-phenolindole dihydrochloride (DAPI). Fluorescence images were captured by an Olympus microscope and analyzed.

### 2.4. Real-Time Quantitative PCR

Total RNA was extracted with TRIzol reagent (Invitrogen, Carlsbad, CA, USA) according to the manufacturer’s established protocol. For gene expression analysis, RNA was converted into cDNA using the SuperScript First-Strand Synthesis System (Invitrogen, Life Technologies, Carlsbad, CA, USA). MALAT1, E-Cadherin (CDH1), N-Cadherin (CDH2), Keratin 10 (KRT10), Zinc Finger E-Box Binding Homeobox 1 (ZEB1), and Alpha-Actin-2 (ACTA2) were amplified using the TaqMan gene expression assay (Applied Biosystems, Waltham, MA, USA). Internal normalization was achieved by using the 18 s housekeeping gene. Samples (n = 5 per group) were amplified in triplicates and results were averaged for each individual sample. The ΔΔCT method was used to calculate relative gene expression. Results are reported as mean ± SD.

### 2.5. Statistical Analysis

Results are expressed as mean ± SD for n = 3 to 5 number of independent experiments. Statistically significant differences in gene expression between two groups was assessed by Student *t*-test. *p* < 0.05 is considered to be statistically significant.

## 3. Results

### 3.1. TGF-β1 and MALAT1 Expression Is Significantly Higher in Diabetic Wounds

**Figure 1:**
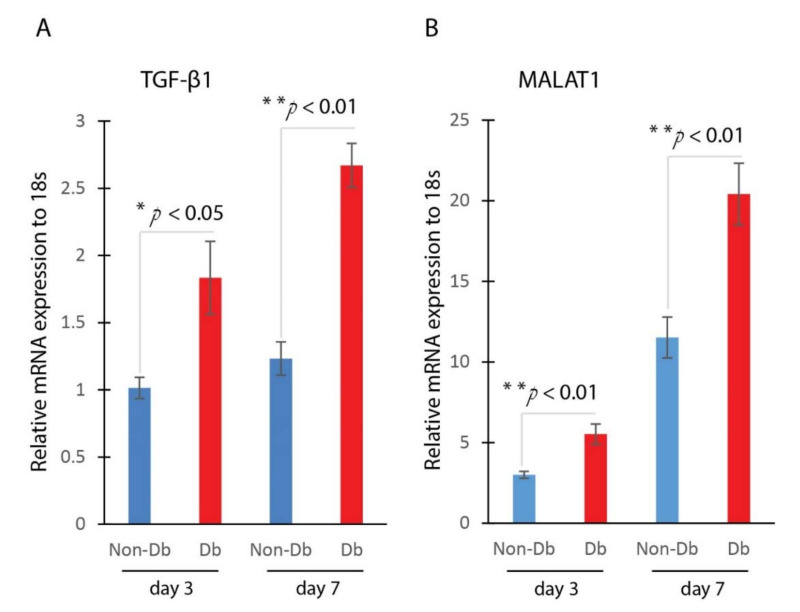
TGF-β1 and MALAT1 expression are significantly higher in diabetic wounds. (**A**) Realtime qPCR analysis of TGF-β1 mRNA expression in mouse diabetic and non-diabetic wounds at day 3 (mean ± SD, n = 5 per group) and day 7 (mean ± SD, n = 5 per group) after injury. (**B**) Realtime qPCR analysis of MALAT1 mRNA expression in mouse diabetic and non-diabetic wounds at day 3 (mean ± SD, n = 5 per group) and day 7 (mean ± SD, n = 5 per group, * *p* < 0.05, ** *p* < 0.01) after injury.

We measured the expression level of genes TGF-β1 and MALAT1 in the wounds. We compared the expression of TGF-β1 and MALAT1 in non-diabetic and diabetic wounds at day 3 and day 7 after injury. Results from Realtime qPCR showed that the expression of TGF-β1 and MALAT1 were significantly up-regulated in diabetic wounds compared to non-diabetic wounds at both day 3 and day 7 after injury (Figure 1). The increased expression of TGF-β1 and MALAT1 in diabetic wounds suggested that they may play a role in the wound-healing process.

### 3.2. TGF-β1 Induces EMT and MALAT1 Expression in HaCaT Cells

**Figure 2:**
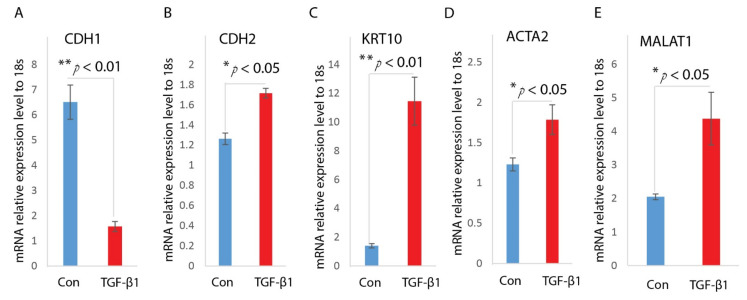
TGF-β1 induces EMT and MALAT1 expression in HaCaT cells. HaCaT cells were treated with TGF-β1(2 ng/mL) after overnight serum starvation. RNAs were isolated and the expressions of CDH1 (**A**), CDH2 (**B**), KRT10 (**C**), and ACTA2 (**D**) were determined by Realtime qPCR. n = 3; mean ± SD; * *p* < 0.05, ** *p* < 0.01 compared with untreated HaCaT cells. (**E**) Levels of MALAT1 in TGF-β1-treated HaCaT cells were determined by Realtime qPCR. 18s was used as an internal control. n = 3; mean ± SD; * *p* < 0.05, ** *p* < 0.01 compared with untreated HaCaT cells.

TGF-β signaling has been proposed as a key factor in regulating EMT of keratinocytes. To understand the higher expression level of TGF-β1 and MALAT1, we analyzed their expression in keratinocyte. We asked whether MALAT1is involved in TGF-β1 induced EMT of HaCaT cells. First, we treated HaCaT cells with TGF-β1 as previously described [26,27]. After incubated with TGF-β1 for 24 h, the HaCaT cells undergo an EMT transition, as confirmed by its morphological change to spindle-shaped cells, decreased expression of epithelial marker CDH1, as well as enhanced expression of mesenchymal markers, including CDH2, ACTA2, and KRT10 (Figure 2A–D).

Next, we measured the MALAT1 levels under TGF-β1 treatment. Realtime qPCR analysis on the treated HaCaT cells indicated that MALAT1 was highly induced under TGF-β1 treatment (Figure 2E). The up-regulation of MALAT1 indicates it may contribute to TGF-β1 mediated EMT of HaCaT cells.

### 3.3. Knockdown of MALAT1 Attenuates the TGF-β1-Induced EMT in HaCat Cells

**Figure 3:**
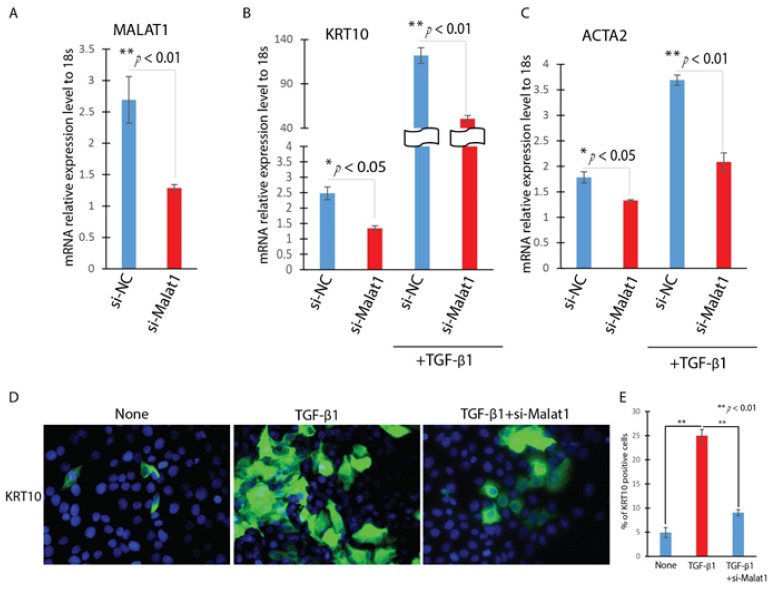
Knockdown of MALAT1 attenuates the TGF-β1 induced EMT in HaCat cells. HaCaT cells were transfected with MALAT1 SiRNA (Si-MALAT1) or negative control SiRNA (Si-NC) and were treated with or without TGF-β1 (2 ng/mL) for 24 h. (**A**). The expression levels of MALAT1 were detected by Realtime qPCR. n = 3; mean ± SD; * *p* < 0.05, ** *p* < 0.01 compared between si-Malat1 and si-NC transfected HaCaT cells. (**B**,**C**). The expression level of EMT-related markers (KRT10 and ACTA2) were detected by Realtime qPCR. (**D**). The expression of KRT10 were detected by immunofluorescence. (**E**). Quantitative analysis of number of KRT10 positive (KRT10 staining) per 20× field. Comparison was performed between PBS, and TGF-β1 treated, or TGF-β1 treated and TGF-β1 treated with si-MALAT1. n = 5; mean ± SD; * *p* < 0.05, ** *p* < 0.01.

We then inquired the role of MALAT1 in TGF-β1-induced EMT in HaCaT cells by knocking down MALAT1. HaCaT cells were transfected with MALAT1-specific siRNA (Si-MALAT1) or a negative control siRNA (Si-NC) after being starved for 16 h. After incubating the cells with TGF-β1 for 24 h, the expression of MALAT1 was then detected by Realtime qPCR. In comparison to Si-NC, transfection with Si-MALAT1 decreased the expression of MALAT1 by more than 60% (Figure 3A). In measuring the expression of EMT-related genes including KRT10 and ACTA2 by Realtime qPCR, we found that knockdown of MALAT1 significantly attenuates TGF-β1 up-regulation of KRT10 and ACTA2 (Figure 3B,C). Furthermore, knockdown of MALAT1 abrogates the TGF-β1 induced morphological change of HaCaT cells and KRT10 positive cells (Figure 3D,E). These results indicated that MALAT1 contributes to the TGF-β1-induced EMT of HaCaT cells.

### 3.4. MALAT1 Regulates EMT-Related Transcription Factor

**Figure 4:**
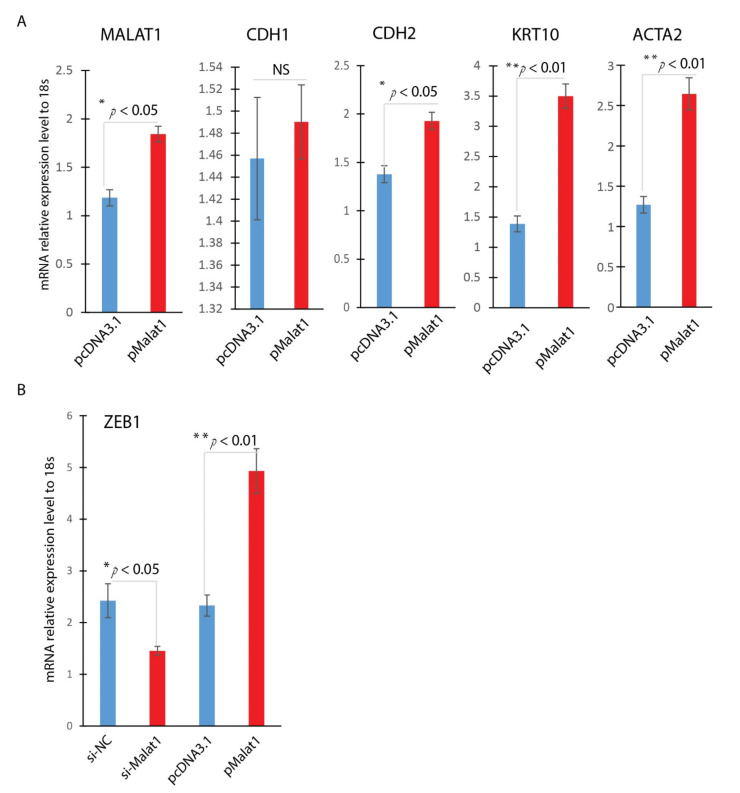
MALAT1 induces ZEB1. Overexpression or knockdown of MALAT1 gene expression was achieved by plasmid transfection or siRNA technology in HaCaT cells. (**A**) Overexpression of Malat1 induces EMT mesenchymal markers (CDH2, KRT10, and ACTA2); (**B**) ZEB1 was significantly induced by MALAT1 overexpression and reduced by MALAT1 knocking down by Realtime qPCR analysis (mean ± SD, n = 3 per group, * *p* < 0.05, ** *p* < 0.01).

In the experiment, we asked the effect of overexpression of MALAT1. Overexpression of MALAT1 was confirmed by Realtime qPCR, indicating a 1.6-fold increase in MALAT1 expression (Figure 4A). Similar to TGF-β1 treated HaCaT Cells, overexpression of MALAT1 enhanced expression of mesenchymal markers, including CDH2, ACTA2, and KRT10 (Figure 4A), and no significant change of CDH1 gene expression. Zinc finger E-box binding homeobox 1 (ZEB1) is a potent EMT activator. Cutaneous ZEB1 is a transcriptional repressor of epithelial genes like E-cadherin [28,29]. Down-regulation of E-cadherin is known to cause EMT. To further confirm the role of MALAT1 in mediating the TGF-β1-induced EMT of HaCaT cells, we detected the expression of EMT-related transcription ZEB1 in HaCaT cells by Realtime qPCR. MALAT1 knocking down was achieved by si-MALAT1transfection while MALAT1overexpression was accomplished by plasmid transfection. As indicated in Figure 4B, knocking down MALAT1 significantly reduced the expression of ZEB1, while overexpression of MALAT1 significantly induced the expression of ZEB1. These results indicated that MALAT1-mediated TGF-β1-induced EMT of HaCaT cells is done, at least partially, through ZEB1, the EMT activator in wound healing.

## 4. Discussion

Chronic wounds are the leading cause for hospital admission in diabetic patients. These chronic wounds are additionally the number one cause of non-traumatic lower extremity amputation [30]. According to the International Federation of Diabetes, every 30 s, a lower limb is lost to a diabetic wound [31]. So far, the effective therapy is unfortunately lacking. The impaired healing of diabetic wounds has been characterized by decreased production of chemokines [32], an abnormal inflammatory response [33], decreased angiogenesis [34], and stalled wound reepithelialization. During the early phase of cutaneous wound healing, stationary keratinocytes will be activated and mobilized toward the wound bed, restoring the epidermal homeostasis. Understanding the factors contributing to this process will enable and allow for findings of novel therapeutic targets or pathways. 

In these studies, we observed that TGF-β1 and MALAT1 were highly expressed in diabetic wounds, prompting us to examine their possible roles in wound healing. In in vitro analysis, human keratinocyte cells, HaCaT was induced to go through EMT under the treatment of TGF-β1, which significantly induced MALAT1. We then knocked down MALAT1 expression in HaCaT and found that knocking down of MALAT1 resulted in significantly decreased expression of mesenchymal marker mRNAs (ACTA2, and KRT10), suggesting that MALAT1 plays a role in TGF-β1 mediated EMT process. Mechanistically, MALAT1 is involved in TGF-β1 mediated EMT through significantly induced ZEB1 expression, a critical transcription factor for EMT. These results suggest a novel link between lncRNA and the EMT in diabetic wounds.

LncRNAs are progressively gaining more attention and have been recognized as important regulators in development and in various human diseases [35,36]. However, the role of lncRNAs in diabetic wound healing has remained largely unclear. MALAT1 is an abundantly expressed nuclear lncRNA that was identified as the first lncRNA associated with metastasis and survival in non-small cell lung cancer [24]. The link between MALAT1 and EMT was documented in various cancer in multiple mechanisms [10,14,15,18,21,37]. Recent studies indicated that MALAT1 has been found to be involved in EMT in non-carcinogenic cells. In endothelial progenitor cells (EPCs), MALAT1 promoted EMT induced by TGF-β1 via competitively binding to miR-145 [38]. In retinal pigment epithelial (RPE), MALAT1 silencing attenuated TGFβ1-induced EMT, migration, and proliferation of RPE cells by activating Smad2/3 signaling [39]. In high glucose (HG)-stimulated HK-2 cells, MALAT1 acts as a sponge RNA for miR-145 to derepress the expression of target gene ZEB2 to induce EMT [40]. These studies indicated that MALAT1 plays an essential role in EMT. Our studies investigate its role in re-epithelialization and EMT in wound healing. Our data is additionally indicative that MALAT1 is required for TGF-β1 mediated keratinocyte EMT.

These findings suggest that MALAT1 may regulate TGF-β1-mediated EMT. The dysregulation of MALAT1 in diabetic wounds may lead to the dysregulation of EMT observed in diabetic wounds. Therefore, MALAT1 may represent a potential therapeutic target to address dysregulated EMT underlying diabetic wound healing impairment.

## Data Availability

Not applicable.

## Abbreviations

EMT	Epithelial Mesenchymal Transition
MALAT1	Metastasis associated lung adenocarcinoma transcript 1
TGF-β1	Transforming growth factor beta 1
CDH1	E-Cadherin
CDH2	N-Cadherin
KRT10	Keratin 10
ACTA2	Alpha-Actin-2
ZEB1	Zinc Finger E-Box Binding Homeobox 1

## References

[1] IDF (2019). IDF Atlas.

[2] Harrington C., Zagari M.J., Corea J., Klitenic J. (2000). A cost analysis of diabetic lower-extremity ulcers. Diabetes Care.

[3] Haensel D., Dai X. (2017). Epithelial-to-mesenchymal transition in cutaneous wound healing: Where we are and where we are heading. Dev. Dyn..

[4] Weber C.E., Li N.Y., Wai P.Y., Kuo P.C. (2012). Epithelial-mesenchymal transition, TGF-β, and osteopontin in wound healing and tissue remodeling after injury. J. Burn. Care Res..

[5] DiStefano J.K. (2018). The Emerging Role of Long Noncoding RNAs in Human Disease. Methods Mol. Biol..

[6] He Z.-Y., Huang M.-T., Cui X., Zhou S.-T., Wu Y., Zhang P.-H., Zhou J. (2021). Long noncoding RNA GAS5 accelerates diabetic wound healing and promotes lymphangiogenesis via miR-217/Prox1 axis. Mol. Cell. Endocrinol..

[7] Hu J., Zhang L., Liechty C., Zgheib C., Hodges M.M., Liechty K.W., Xu J. (2020). Long Noncoding RNA GAS5 Regulates Macrophage Polarization and Diabetic Wound Healing. J. Investig. Dermatol..

[8] Peng W.X., He P.X., Liu L.J., Zhu T., Zhong Y.Q., Xiang L., Peng K., Yang J.J., Xiang G.D. (2021). LncRNA GAS5 activates the HIF1A/VEGF pathway by binding to TAF15 to promote wound healing in diabetic foot ulcers. Lab. Investig..

[9] Zgheib C., Hodges M.M., Hu J., Liechty K.W., Xu J. (2017). Long non-coding RNA Lethe regulates hyperglycemia-induced reactive oxygen species production in macrophages. PLoS ONE.

[10] Chen M., Xia Z., Chen C., Hu W., Yuan Y. (2018). LncRNA MALAT1 promotes epithelial-to-mesenchymal transition of esophageal cancer through Ezh2-Notch1 signaling pathway. Anti-Cancer Drugs.

[11] Xu S., Sui S., Zhang J., Bai N., Shi Q., Zhang G., Gao S., You Z., Zhan C., Liu F. (2015). Downregulation of long noncoding RNA MALAT1 induces epithelial-to-mesenchymal transition via the PI3K-AKT pathway in breast cancer. Int. J. Clin. Exp. Pathol..

[12] Zhou X., Liu S., Cai G., Kong L., Zhang T., Ren Y., Wu Y., Mei M., Zhang L., Wang X. (2015). Long Non Coding RNA MALAT1 Promotes Tumor Growth and Metastasis by inducing Epithelial-Mesenchymal Transition in Oral Squamous Cell Carcinoma. Sci. Rep..

[13] Zhao C., Ling X., Xia Y., Yan B., Guan Q. (2021). The m6A methyltransferase METTL3 controls epithelial-mesenchymal transition, migration and invasion of breast cancer through the MALAT1/miR-26b/HMGA2 axis. Cancer Cell Int..

[14] Radwan A., Shaker O., El-Boghdady N., Senousy M. (2021). Association of MALAT1 and PVT1 Variants, Expression Profiles and Target miRNA-101 and miRNA-186 with Colorectal Cancer: Correlation with Epithelial-Mesenchymal Transition. Int. J. Mol. Sci..

[15] Sun Z., Ou C., Liu J., Chen C., Zhou Q., Yang S., Li G., Wang G., Song J., Li Z. (2019). YAP1-induced MALAT1 promotes epithelial-mesenchymal transition and angiogenesis by sponging miR-126-5p in colorectal cancer. Oncogene.

[16] Wang Y., Zhou Y., Yang Z., Chen B., Huang W., Liu Y., Zhang Y. (2017). MiR-204/ZEB2 axis functions as key mediator for MALAT1-induced epithelial–mesenchymal transition in breast cancer. Tumor Biol..

[17] Latorre E., Carelli S., Raimondi I., D’Agostino V., Castiglioni I., Zucal C., Moro G., Luciani A., Ghilardi G., Monti E. (2016). The Ribonucleic Complex HuR-MALAT1 Represses CD133 Expression and Suppresses Epithelial-Mesenchymal Transition in Breast Cancer. Cancer Res..

[18] Sun R., Qin C., Jiang B., Fang S., Pan X., Peng L., Liu Z., Li W., Li Y., Li G. (2016). Down-regulation of MALAT1 inhibits cervical cancer cell invasion and metastasis by inhibition of epithelial–mesenchymal transition. Mol. BioSyst..

[19] Dong N. (2019). Long Noncoding RNA MALAT1 Acts as a Competing Endogenous RNA to Regulate TGF-*β*2 Induced Epithelial-Mesenchymal Transition of Lens Epithelial Cells by a MicroRNA-26a-Dependent Mechanism. Biomed. Res. Int..

[20] Lu L., Luo F., Liu Y., Liu X., Shi L., Lu X., Liu Q. (2015). Posttranscriptional silencing of the lncRNA MALAT1 by miR-217 inhibits the epithelial–mesenchymal transition via enhancer of zeste homolog 2 in the malignant transformation of HBE cells induced by cigarette smoke extract. Toxicol. Appl. Pharmacol..

[21] Shen L., Chen L., Wang Y., Jiang X., Xia H., Zhuang Z. (2014). Long noncoding RNA MALAT1 promotes brain metastasis by inducing epithelial-mesenchymal transition in lung cancer. J. Neuro-Oncol..

[22] Zhang Y., Qu L., Ni H., Wang Y., Li L., Yang X., Wang X., Hou Y. (2020). Expression and function of lncRNA MALAT1 in gestational diabetes mellitus. Adv. Clin. Exp. Med..

[23] Liu S.X., Zheng F., Xie K.L., Xie M.R., Jiang L.J., Cai Y. (2019). Exercise Reduces Insulin Resistance in Type 2 Diabetes Mellitus via Mediating the lncRNA MALAT1/MicroRNA-382-3p/Resistin Axis. Mol. Ther. Nucleic Acids.

[24] Abdulle L.E., Hao J.-L., Pant O.P., Liu X.-F., Zhou D.-D., Gao Y., Suwal A., Lu C.-W. (2019). MALAT1 as a Diagnostic and Therapeutic Target in Diabetes-Related Complications: A Promising Long-Noncoding RNA. Int. J. Med. Sci..

[25] Liu J.Y., Yao J., Li X.M., Song Y.C., Wang X.Q., Li Y.J., Yan B., Jiang Q. (2014). Pathogenic role of lncRNA-MALAT1 in endothelial cell dysfunction in diabetes mellitus. Cell Death Dis..

[26] Liarte S., Bernabé-García Á., Nicolás F.J. (2020). Human Skin Keratinocytes on Sustained TGF-β Stimulation Reveal Partial EMT Features and Weaken Growth Arrest Responses. Cells.

[27] Miyake Y., Nagaoka Y., Okamura K., Takeishi Y., Tamaoki S., Hatta M. (2021). SNAI2 is induced by transforming growth factor-β1, but is not essential for epithelial-mesenchymal transition in human keratinocyte HaCaT cells. Exp. Ther. Med..

[28] Liu Y., El-Naggar S., Darling D.S., Higashi Y., Dean D.C. (2008). Zeb1 links epithelial-mesenchymal transition and cellular senescence. Development.

[29] Singh K., Sinha M., Pal D., Tabasum S., Gnyawali S.C., Khona D., Sarkar S., Mohanty S.K., Soto-Gonzalez F., Khanna S. (2019). Cutaneous Epithelial to Mesenchymal Transition Activator ZEB1 Regulates Wound Angio-genesis and Closure in a Glycemic Status-Dependent Manner. Diabetes.

[30] Reiber G.E., Vileikyte L., Boyko E., Del Aguila M., Smith D.G., Lavery L.A., Boulton A.J. (1999). Causal pathways for incident lower-extremity ulcers in patients with diabetes from two settings. Diabetes Care.

[31] Bakker K., van Houtum W.H., Riley P.C. (2005). 2005: The International Diabetes Federation focuses on the diabetic foot. Curr. Diabetes Rep..

[32] Jude E.B., Blakytny R., Bulmer J., Boulton A.J.M., Ferguson M.W.J. (2002). Transforming growth factor-beta 1, 2, 3 and receptor type I and II in diabetic foot ulcers. Diabet. Med..

[33] Fahey T.J., Sadaty A., Jones W.G., Barber A., Smoller B., Shires G.T. (1991). Diabetes impairs the late inflammatory response to wound healing. J. Surg. Res..

[34] Martin A., Komada M.R., Sane D.C. (2003). Abnormal angiogenesis in diabetes mellitus. Med. Res. Rev..

[35] Rao A.K.D.M., Rajkumar T., Mani S. (2017). Perspectives of long non-coding RNAs in cancer. Mol. Biol. Rep..

[36] Wu T., Du Y. (2017). LncRNAs: From Basic Research to Medical Application. Int. J. Biol. Sci..

[37] Peng C., Wang Y., Ji L., Kuang L., Yu Z., Li H., Zhang J., Zhao J. (2021). LncRNA-MALAT1/miRNA-204-5p/Smad4 Axis Regulates Epithelial-Mesenchymal Transition, Proliferation and Migration of Lens Epithelial Cells. Curr. Eye Res..

[38] Xiang Y., Zhang Y., Tang Y., Li Q. (2017). MALAT1 Modulates TGF-β1-Induced Endothelial-to-Mesenchymal Transition through Downregulation of miR-145. Cell. Physiol. Biochem..

[39] Yang S., Yao H., Li M., Li H., Wang F. (2016). Long Non-Coding RNA MALAT1 Mediates Transforming Growth Factor Beta1-Induced Epithelial-Mesenchymal Transition of Retinal Pigment Epithelial Cells. PLoS ONE.

[40] Liu B., Qiang L., Wang G.D., Duan Q., Liu J. (2019). LncRNA MALAT1 facilities high glucose induced endothelial to mesenchymal transition and fibrosis via targeting miR-145/ZEB2 axis. Eur. Rev. Med. Pharmacol. Sci..

